# Clinical application of CNS injury biomarkers in anesthesia and intensive care

**DOI:** 10.1016/j.bjane.2025.844690

**Published:** 2025-10-20

**Authors:** Katarzyna Prus, Jamel Ortoleva, Federico Bilotta

**Affiliations:** a University Clinical Hospital No 4, Department of Neurology, Stroke and Early Poststroke Rehabilitation, Lublin, Poland; b Boston University Chobanian & Avedisian School of Medicine, Department of Anesthesiology, Boston, USA; c Tor Vergata University, Department of Anesthesiology and Intensive Care, Rome, Italy

## Introduction

Central nervous system (CNS) injury as a consequence of trauma, ischemia, hemorrhage, or critical illness is a significant medical problem affecting millions of patients worldwide. Furthermore, all intensive care and surgical patients are at risk of neurological sequelae, including not only organic brain damage such as stroke or hemorrhage, but also postoperative delirium (POD) or postoperative cognitive disorders (POCD). Despite recent progress in neuroimaging and clinical management, CNS injuries remain the leading cause of mortality and disability in all age groups.^1^ We are still lacking refined laboratory measures that can be implemented in everyday practice to facilitate diagnostic workups, prognostication, and monitoring for reversible sequelae that cause secondary injury.

The ideal biomarker of CNS injury should possess similar characteristics to other diagnostic markers, such as cardiac troponins. The biomarker should be CNS-specific, have high sensitivity and specificity for diagnostic and prognostic purposes, yield rapid results, and be widely accessible – this includes easily obtainable sampling material as well as economic efficiency of the measurements. Biomarkers may enable the monitoring of CNS-related complications and the prediction of future outcomes after brain injury and critical illness. They could also facilitate preoperative evaluation of patients who are potentially susceptible to postoperative neurological dysfunction, such as POD and POCD. Recently, several agents have been proposed as potential biomarker candidates for assessing CNS injury. These markers reflect pathological processes following brain tissue injury, such as neuronal and glial damage, axonal injury, neuroinflammation, and increased blood-brain barrier (BBB) permeability. This article provides an overview of available research data on the clinical utility of potential CNS biomarkers in anesthesia and intensive care.

## Possible clinical application of CNS biomarkers in anesthesiology and intensive care

### Perioperative Assessment

Preoperative assessment and postoperative monitoring for neurological complications are essential for optimal patient outcomes. In the perioperative setting, CNS biomarkers could facilitate rapid detection and evaluation of patients presenting with postoperative neurological dysfunction. Neurological sequelae are common in the postoperative period, and specific patient groups may be at higher risk of developing them. Biomarkers can supplement other diagnostic modalities, such as neuroimaging, EEG, or transcranial Doppler, in monitoring for potential neurological complications following surgical procedures. Numerous potential biomarker candidates were identified that may provide insights into neuronal damage, neuroinflammation, or secondary brain ischemia following operative treatment. Furthermore, there is data on the potential clinical utility of biomarkers in monitoring the neurological consequences of anesthesia.

During the acute postoperative phase, surgery-induced tissue injury results in the release of cytokines and chemokines, which increase the permeability of the BBB and lead to further vascular and neuronal damage.^2^ Numerous studies have proven that this process is associated with elevated levels of CNS-derived proteins, including glial fibrillary acidic protein (GFAP), neurofilament light (NfL), tau protein, and S100B protein, in both blood and CSF samples of patients following different types of invasive procedures.^3^ Some groups of patients may be more prone to surgery-induced CNS injury. Postoperative levels of S100B were reported to be higher in elderly patients and ApoE 4 carriers, suggesting that age and genetic susceptibility may influence the serum concentrations of S100B in cases of Alzheimer's Disease (AD), intracerebral hemorrhage, head trauma, and brain hypoperfusion during surgeries with cardiopulmonary bypass. Research on POD and its relationship with CNS biomarkers has reported that higher serum levels of IL-6, CRP, S100B, and NfL, as well as altered serum expression of selected miRNAs, are associated with POD.^4^^,^^5^ Perioperative biomarker assessment may enable the identification of patients at risk of delirium and POCD, along with predicting long-term patient outcomes.

### Neurological complications of critical illness

Intensive care patients are particularly vulnerable to neurological complications such as delirium, cognitive dysfunction, seizures, encephalopathy, or delayed cerebral ischemia. There are reports on the prognostic value of selected CNS-derived proteins in critical care patients. GFAP, S100B, NfL, neuron-specific enolase (NSE), and Ubiquitin Carboxyl-terminal Hydrolase L1 (UCHL-1) have been investigated as prognostic markers for critically ill patients with various comorbidities.

The potential prognostic utility of CNS biomarkers has been confirmed in several studies involving both adult and pediatric patients after cardiac arrest. Elevated CNS protein levels following cardiac arrest can result from multiple mechanisms, including neuronal apoptosis and BBB disruption. NSE has been confirmed to show an ability to discriminate between survivors and non-survivors of cardiac arrest, and levels of NSE at 48 h post-admission or 72 h post-cardiac arrest are associated with a 90-day outcome. Guidelines recommend NSE level assessment as part of post-cardiac arrest care.^6^ The latest meta-analysis on the utility of brain biomarkers in predicting survival and neurological outcomes in pediatric patients confirmed that NSE is correlated with prognosis and neurological outcomes in this population.

Furthermore, UCHL-1 and GFAP demonstrated promising potential for stratifying early outcomes.^7^ Studies on NfL have similarly shown promising data on prognostication in out-of-hospital cardiac arrest (OHCA). In a prospective study, serum NfL levels 1–3 days after OHCA were correlated with worse neurological outcomes at 6 months, and this prognostic performance exceeded that of standardized neuromonitoring techniques and other biomarkers.^8^ Biomarker studies analyzing their potential prognostic utility were performed in patients undergoing Extracorporeal Membrane Oxygenation (ECMO). It is reported that higher serum levels of selected biomarkers (GFAP, NSE, S100B) are correlated with the extent of brain injury and mortality. They were also independently associated with survival and functional outcomes in ECMO patients.^9^

Research data indicate that assessing CNS injury biomarkers may help predict cognitive disorders in the course of critical illness. A prospective study of a large group of patients with respiratory failure on mechanical ventilation found that NfL concentration, measured early in the course of hospitalization, was associated with a clinical diagnosis of delirium.^4^ On the other hand, some CNS-derived proteins may play a protective role; studies have shown that elevated levels of brain-derived neurotrophic factor (BDNF) and UCHL-1 early after ICU admission are associated with a decreased risk of delirium in critical care patients.^4^^,^^10^ Some pharmaceuticals may enhance this effect. Dexmedetomidine, which exerts neuroprotective properties mediated by BDNF, was proven to reduce the incidence rate of POD in neurosurgery patients.^11^

### Traumatic brain injury assessment and prognostication

Primary brain injuries account for a significant portion of patients hospitalized in the ICU. CNS biomarker assessment can assist in prognostication and crucial therapeutic decisions. The CNS biomarkers have been extensively studied in traumatic brain injury (TBI) patients for use in diagnostic workups, monitoring, and prognostication of long-term outcomes. GFAP, UCHL-1, and S100B are already established in clinical practice to exclude the presence of lesions in CT scans in case of mild to moderate head trauma.^12^^,^^13^ These biomarkers are available for quick, bedside assessments, helping to reduce the need for high-risk transportation, imaging, and radiation in this patient group. Recently, more agents have been investigated for use as both diagnostic and prognostic markers of brain damage, mainly in correlation with clinical grading scales. Studies reported that S100B and NSE levels in conjunction with the APACHE II calculation are efficient predictors of compromised outcome among critically ill patients with primary brain injuries.^14^ Another study by Ito et al. suggests that in TBI patients, levels of growth differentiation factor 15 (GDF-15) are correlated with Sequential Organ Failure Assessment scores.^15^

Recent studies have demonstrated the diagnostic and prognostic potential of miRNA profiling in TBI patients. Researchers have reported altered expression of selected miRNAs following TBI.^16^ Data prove that combining the use of miRNAs, CNS-derived proteins, and markers of inflammation can enhance the specificity and sensitivity of prognostic assessments.^17^ New reports highlight the potential utility of urine and saliva samples for detecting biomarkers of CNS injury. UCHL-1 has been suggested as a promising diagnostic marker in urine samples of patients with TBI,^18^ and miRNA profiling of saliva demonstrated elevated expression of specific miRNAs in a population of pediatric patients following brain concussion.^19^

### Stroke

Stroke, including acute ischemia, intracerebral hemorrhage (ICH), and subarachnoid hemorrhage (SAH), is a CNS injury that often results in a critical state. It remains one of the most important causes of disability and mortality in adult patients. Despite ongoing progress in neuroimaging and reperfusion treatment, a universal biomarker for ischemic or hemorrhagic brain injury remains elusive. CNS-derived proteins, miRNA, and inflammatory agents proposed as potential acute ischemic stroke (AIS) biomarkers are reported to be associated with specific pathological features in the course of stroke – neuronal death, increased BBB permeability, neuroinflammation, delayed cerebral ischemia, or secondary hemorrhagic transformation. Research suggests that serum GFAP may be used to differentiate between AIS and ICH, provide insights into the time from symptom onset, and the extent of the ischemic lesion.^20^ There is data on the correlation of other CNS-derived proteins and the clinical severity of AIS. It is reported that elevated levels of β-synuclein, NfL, and GFAP are associated with higher NIHSS scores and lower Alberta Stroke Program CT Score on admission.^21^

There is growing interest in the potential application of miRNA profiling in stroke diagnostics and prognostication. Research brings interesting data on the correlation between the expression of selected miRNAs (miR-125b-5p, miR-143, miR-146b, miR-218, miR-21, miR-93, miR-29b, miR-126, and miR-130) and critical clinical features of stroke, such as the volume of the ischemic lesion, systemic inflammation, or neurological deficit.^22^ It is also suggested that miRNA assessment may bring insights into the evaluation of the efficacy of reperfusion treatment of AIS patients with large vessel occlusion.^23^ Possible use of miRNA biomarkers was also confirmed for hemorrhagic stroke. A systematic review on miRNA signatures in ICH patients revealed a potential role for miRNAs as biomarkers for the early detection and differentiation of ICH.^24^ Assessment of miRNA expression in aneurysmal SAH patients revealed an upregulation of miRNAs during vasospasm, suggesting a potential for early detection and monitoring for delayed cerebral ischemia in this patient group.^25^

The future consequences of stroke may be strongly correlated not only with complications and comorbidities, but also with medical procedures that may affect the perfusion, oxygenation, and metabolism of brain tissue. Growing interest in biomarkers of reperfusion after endovascular treatment highlights the importance of selecting the optimal anesthetic method for mechanical thrombectomy in AIS and improving perioperative care to reduce the extent of brain injury and enhance patient outcomes.^26^^,^^27^

## Limitations and Challenges

The implementation of blood biomarkers for CNS injury still presents considerable challenges, including pre-analytical and analytical standardization, comorbidities, and the diverse demographics of the studied population.

One of the main confounding factors is the fact that most agents proposed as biomarkers for CNS injury are not specific to the nervous system. S100B can be found in non-nervous tissues, such as skin, muscle, and bone, and its elevated level in blood samples may result from extracranial injuries like burns or fractures.^28^ GFAP serum concentration can also be higher in the course of inflammatory and degenerative diseases, such as inflammatory bowel disease, hepatic fibrosis, Parkinson's disease, or following complicated surgical procedures.^29^ Moreover, the correlation between CNS and blood concentration of many biomarkers remains unclear. In the case of S100B, studies demonstrate that CSF levels may be more predictive of outcome than serum or plasma levels. An increase in GFAP blood level has been well-documented in ABI with transient blood-brain barrier disruptions. This aspect suggests that the release of these biomarkers during CNS injury may be more closely correlated with impaired BBB function than with intraparenchymal pathology. Emerging research suggests that miRNAs have high specificity for tissue or cell types, and their expression may also vary according to disease progression or therapy responsiveness. Data indicate that miRNA can cross the BBB and are remarkably stable in peripheral biofluids, even under extreme conditions, which makes them potentially interesting biomarker candidates.^30^

The standardization of CNS injury biomarker measurements may additionally be affected by demographic factors and comorbidities. Research indicates that CSF levels of S100B are significantly correlated with age and gender, with higher levels observed in women and older individuals.^31^ Even in the case of the most standardized tests, such as chemiluminescence ELISA for GFAP/UCHL-1 tandem assessment, there is a significant variability influenced by age, genetic ethnicity, and systemic trauma. This fact underscores the need for age-stratified reference ranges and recalibration across diverse ethnic populations to ensure the accurate interpretation of results.^32^

Validated and standardized tests for detecting CNS injury biomarkers are crucial for the widespread adoption and implementation in clinical practice. To acquire objective data, not only assessment methods, but also sample collection timing and indications for sample handling need to be systematized. Only reproducible and consistent results obtained across laboratories and assay kits can increase the confidence of both users and regulatory agencies in the future widespread use of blood biomarkers in ABI management.^33^

## Conclusions and future perspectives

Biomarkers have the potential to play a crucial role in the diagnosis, prognosis, and treatment of neurocritical patients. They may provide an objective and measurable assessment of CNS pathology. Clinical applications in various areas of anesthesia and intensive care, such as preoperative assessment, monitoring postoperative neurological complications, and early detection of brain pathologies, could significantly improve management. Despite multiple studies on possible indicative agents, the translation of biomarkers from laboratory findings to clinical practice is not always feasible. Before CNS biomarkers can be successfully implemented into routine clinical evaluation, further research is needed to develop standardized assessment methods and address the clinical challenges associated with their use. Furthermore, it is necessary to assess the implementation costs, potential budget impacts, and long-term effectiveness of biomarkers in everyday clinical practice. Future randomized validation trials with precisely designed protocols are crucial for determining the diagnostic and prognostic accuracy of proposed brain injury biomarkers and evaluating their potential role in the medical management of patients with CNS disorders.

Editor: Liana Azi

## Conflicts of interest

The authors declare no conflicts of interest.
